# Adapting family-based treatment for adolescent anorexia nervosa delivered in the home: A novel approach for improving access to care and generalizability of skill acquisition

**DOI:** 10.1186/s40337-023-00850-8

**Published:** 2023-08-05

**Authors:** Andrea B. Goldschmidt, Christina C. Tortolani, Erin C. Accurso, Eva-Molly Petitto Dunbar, Amy H. Egbert, Deidre Donaldson, Abigail A. Donaldson

**Affiliations:** 1 Department of Psychiatry, Western Psychiatric Institute and Clinic, University of Pittsburgh School of Medicine, Pittsburgh, PA USA; 2 Department of Counseling, Educational Leadership, and School Psychology, Rhode Island College, Providence, RI USA; 3 Department of Psychiatry and Human Behavior, Warren Alpert Medical School of Brown University/The Miriam Hospital, Providence, RI USA; 4 Department of Psychiatry and Behavioral Sciences, Weill Institute for Neurosciences, University of California, San Francisco, CA USA; 5 Department of Psychology, University of Rhode Island, Kingstown, RI USA; 6 Department of Psychological Sciences, University of Connecticut, Connecticut Storz, USA; 7 Department of Family Medicine, Warren Alpert Medical School of Brown University/Gateway Healthcare, Providence, RI USA; 8 Department of Pediatrics, Warren Alpert Medical School of Brown University/Rhode Island Hospital, Providence, RI USA

**Keywords:** Family-based treatment, Anorexia nervosa, Restrictive eating, Home-based, Accessibility, Adaptation

## Abstract

**Background:**

Anorexia nervosa (AN) is a serious mental illness associated with high rates of morbidity and mortality. Family-based treatment (FBT) is a well-established treatment for adolescent AN, yet it is underutilized in community settings and is unavailable to many families, particularly those from lower income and racial and ethnic minority backgrounds. Furthermore, some families do not respond optimally to FBT, possibly because of challenges translating skills acquired in office-based treatment settings to naturalistic settings. Home-based treatment could reduce barriers to access and enhance generalization of newly learned treatment skills. Home-based models demonstrate initial feasibility, acceptability, and efficacy for adolescent AN, however, FBT principles have yet to be applied as a stand-alone intervention in a home-based level of care. This paper describes the rationale for and process of adapting FBT principles/interventions to improve fit within a home-based model delivered in the context of community mental health, and discusses potential strengths and opportunities associated with this approach.

**Results:**

Adaptations were made through consultation with collaborating community agencies and were guided by the complex interventions framework. The primary modifications included: (1) altered dose; (2) multiple family meals; (3) additional support for meal preparation and supervision; (4) clinician attendance at medical appointments; (5) cultural adaptation; and (6) introduction of distress tolerance and emotion regulation skills.

**Conclusions:**

Implementing FBT in the home may present one promising and novel approach to enhance engagement and treatment outcomes for adolescents with restrictive eating disorders, particularly those who are underserved, but evaluation of efficacy/effectiveness is needed.

## Background

Anorexia nervosa (AN) and its subthreshold/atypical variants affect 3–4% of individuals in the community [1] and are associated with decreased quality of life, high caregiver burden, and increased mortality [2]. Rates and severity of these disorders have markedly increased in the context of COVID-19, accompanied by predictable increases in healthcare utilization [3]. There are several efficacious interventions for adolescent AN [4], including family-based treatment (FBT). FBT is a time-limited, behavioral intervention in which caregivers are charged with re-feeding their child with AN to normalize eating and weight status [5]. More than 50% of recipients achieve weight restoration upon completion of FBT, and effects on eating and weight are largely sustained or improved over follow-up [2]. However, like many evidence-based treatments, FBT is often inaccessible due to cost, geographic constraints, stigma, and other challenges [6]. These constraints may be particularly salient for families from lower income and racial and ethnic minority backgrounds, who despite being affected by eating disorders at rates that are approximately proportionate to their representation in the population [7, 8], frequently underutilize treatment [9]. Therefore, while FBT is a promising first-line treatment approach for adolescent AN, it requires adaptations to improve access for families, many of whom would otherwise receive suboptimal or no care.

Implementation of FBT is low outside of specialty research and private practice settings [10]. Indeed, there were only 64 FBT-certified clinicians in the U.S. in 2021, with several states lacking a single certified provider (11; although, of note, these estimates may underrepresent broader community practice of FBT given the costs associated with certification). Further, this small number of FBT clinicians reside in densely populated urban areas and largely serve the privileged few who can pay out-of-pocket costs (with only one-third of certified providers contracted with at least one private health insurance plan, and only three contracted with Medicaid; [11]). The gap in access to FBT, which disproportionately impacts families living in rural regions and those with lower incomes, may lead to overreliance on medical care (including inpatient services) and increased risk for developing a chronic course of illness requiring costly treatment [12].

Implementation science methods are needed to improve availability and uptake of FBT in community-based settings [13]. Adaptation is a process of thoughtful and deliberate modification to the design or delivery of an intervention to improve its fit or effectiveness in a particular context [14, 15]. Adaptations that optimize the fit of evidence-based interventions for patients/families can improve engagement and clinical outcomes, especially with minority populations [16, 17]. Modifications are often made by providers in real-world implementation, but proactive planned adaptation is likely more advantageous in sustainment of evidence-based practices [18–20]. This paper describes the process of adapting FBT to improve its fit when delivered by master’s-level, non-specialist mental health providers serving a largely lower-income, racially and ethnically diverse, and high acuity population of adolescents with AN-spectrum disorders in two community-based mental health settings offering home-based services in Rhode Island. The adaptation process was informed by the complex interventions framework [21, 22] and the description of adaptations was guided by the Framework for Reporting Adaptations and Modifications-Enhanced (FRAME; [23]).

### Rationale for applying FBT principles in the home

Common barriers to seeking eating disorders treatment include travel and transportation-related constraints, mental health stigma, and conflicting demands of other family commitments (e.g., needs of other dependent children in the home; [24]). Additionally, community partners often cite difficulties that families experience when participating in office-based care, particularly those with fewer resources. As a result, a large number of agencies offer wraparound services in the family’s own environment (e.g., home) to reduce barriers to accessing care. Home-based treatment eliminates travel for families, minimizes mental health stigma and discomfort through providing services outside of a traditional healthcare setting, and decreases burden on families who need to schedule during non-business hours or require alternative caregivers for other dependent children when attending appointments outside the home [25].

Telehealth addresses several barriers for some populations [26], and has seen tremendous growth in uptake as a function of the COVID-19 pandemic [27]. However, our community partners described additional barriers faced by both providers and families when engaging in telehealth, including lack of consistent access to a device and/or WiFi, difficulties seeing all participating family members on the screen, limited ability to survey aspects of the home environment that might inform delivery of FBT, and increased distractions that cannot be easily managed when the clinician is in a separate physical space (e.g., adolescents using treatment-interfering behaviors that inhibit continuation of sessions with caregivers alone). Further, families and clinicians often describe struggling with engagement, therapeutic alliance, and appropriate management of medical/psychiatric risk during virtual treatment [28]. Preference for telehealth over face-to-face therapy has waned markedly as the pandemic has receded, and as a result of community partner input regarding feasibility and acceptability, telehealth was ruled out as a permanent adaptation.

Home-based treatment has been well-established in Europe to provide interim care when higher levels of care are unavailable and decrease hospitalization (e.g., [29–33]). In the U.S., it is available in ≥ 43 states, and is covered by Medicaid and 7 of the top 8 U.S. commercial insurance plans. In 2013, the Centers for Medicaid and Children’s Health Insurance Program (CHIP) Services released a bulletin encouraging use of home- and community-based mental health services for youth based on reduced costs of care, improved school attendance/performance and behavioral/emotional functioning, increased stability in living situations, and improved caregiver work attendance [34]. Home-based adaptations of evidence-based treatments for other psychiatric disorders (e.g., obsessive–compulsive disorder) have demonstrated initial efficacy [35], and early investigations suggest that adding home-based treatment to FBT [36] or implementing it within a stepped-care model to reduce inpatient hospitalization [37] is feasible, acceptable, and efficacious in improving AN symptoms.

In addition to practical benefits, home-based treatment may also address heterogeneity in treatment response [2], which may in part be a function of difficulties generalizing new skills learned in specific healthcare settings (e.g., clinician’s office) to day-to-day, naturalistic settings (e.g., home environment). Translational research suggests that new learning is context-specific [38], such that the setting (and other contextual factors) in which new learning occurs becomes a cue associated with newly learned skills/behaviors. Repetition of these behaviors is facilitated when contextual cues associated with new learning are present at the time of repetition [39]. Although much of this research has been conducted in the addictions field, with limited research applied specifically to AN or other eating disorders, parallels between these types of disorders (e.g., compulsive nature of behaviors, shared neurobiology; [40]) suggest that such a theoretical framework could be usefully applied to understanding AN and its management as well. Indeed, the importance of context-dependent learning may be especially salient when attempting to extinguish habitual behaviors, such as those that characterize AN [41], as habits are sensitive to changes in context [42]. Interventions designed to evoke newly learned, adaptive responses to AN behaviors (e.g., parental insistence that the adolescent take “one more bite” than planned) may be more effective when taught in a variety of naturalistic contexts/settings, including those that are familiar (e.g., home setting).

We propose that a home-based care model may provide a viable framework in which to apply FBT interventions to improve its reach, feasibility, and fit. Specific advantages of this approach include the ability to observe aspects of the home environment that would not otherwise be evident; opportunities for in vivo psychoeducation and modeling in meal planning, preparation, and supervision; ability to provide support in school and community settings; and potential to include family members who live in the home but are unable or unwilling to attend office-based outpatient FBT (e.g., multigenerational caregivers). The core functions and forms framework posits that the core functions of a complex behavioral health intervention are structural and procedural goals to reach the intervention goals, and that activities carried out to support each core function (i.e., forms) need to be customized and tailored to local settings and patient populations [43]. Optimally leveraging each of these opportunities while adhering to the core tenets of FBT required adaptations to fit the needs of the community agencies, clinicians, and families. For example, interventions designed to empower caregivers in refeeding efforts may vary for families with limited resources or social privileges (e.g., due to language constraints, immigration status, and other sociocultural factors impacting capacity to advocate for their children in healthcare settings). Below, we describe FBT’s core functions [43] and the process through which adaptations to the forms were developed.

## Methods

Over the last seven years, our group has been advancing eating disorder treatment dissemination in two community-based behavioral health agencies in Rhode Island (Gateway Healthcare and The Providence Center) that offer home-based services [44]. This work was undertaken in the context of a pilot open trial of home-based FBT [45], accompanying qualitative research on provider experiences delivering/supporting delivery of FBT in the home setting [46], and an ongoing effectiveness-implementation randomized controlled trial (RCT) of home-based FBT versus home-based “treatment as usual” [47]. In these settings, home-based treatment is conceptualized as a short, intense “burst” of treatment (e.g., 2–3 two-hour sessions per week over 10–16 weeks) for youth with acute illness presentations who require a higher or more intense dose of services to remain stable in their home and community. Thus, home-based treatment is designed to provide an intermediate level of care between outpatient and hospital-based treatment, or an alternative to higher levels of care (e.g., for those on waiting lists for higher levels of care or with limited insurance coverage; [48]). While families may self-refer, referrals are often made during the hospital stay for continuity of aftercare. Treatment primarily occurs in the home and community contexts (e.g., school, other healthcare settings) and typically includes some combination of master's-level clinicians, case managers, and/or behavior specialists. Because patients presenting for home-based treatment all have acute needs to qualify for this intensive level of support, yet must be medically stable for outpatient treatment, the initial dose is similar across patients but may be tapered depending on response to treatment (alternatively, as with any outpatient treatment, patients may be stepped up to a higher level of care as medical and psychiatric needs dictate).

As part of our implementation efforts, we have undergone an iterative and transactional process of adapting FBT interventions for the home. These adaptations were introduced to increase reach, improve feasibility, and optimize fit both within the structure of the agencies, and with the families presenting for treatment, who were frequently lower income, racially and ethnically diverse (predominantly Hispanic or Latino, consistent with state census data), and diagnostically complex with high illness acuity (including approximately 50% of referrals recently discharged from a higher level of care). This process included first familiarizing ourselves with the patients, providers, and settings to which we aimed to transport FBT, and training the providers in traditional outpatient FBT (via 1.5 day live workshops led by experts in FBT, and provision of the FBT manual). These activities led us to operationalize and integrate empirically-based and stakeholder-informed adaptations into manualized FBT to improve fit. Prior to offering FBT in the home, we consulted with team leaders, supervisors, and clinicians at the community agencies (including multicultural and multilingual team members who assisted with decision-making around cultural adaptations), as well as an author of the FBT manual [5]. We gained critical knowledge from this process, including an understanding that manualized FBT did not meet the needs of families with limited resources (e.g., transportation difficulties, minimal time and support to focus on refeeding) who typically present to community agencies. Together with team leaders, supervisors, and clinicians within these agencies, we collaboratively decided to adapt FBT to be delivered in the home setting with the goals of improving engagement, retention, and opportunities for support. A collaborative decision was made to intensify the treatment dose, consistent with a home-based treatment model, to better support caregivers engaging in refeeding.

Based on this formative work, we determined that the following interrelated modifications were necessary to improve the fit of FBT in a home-based treatment model: (1) altered dose of treatment (~ 30–40 h of treatment over 12–16 weeks vs. 16–20 h of treatment over 6–12 months); (2) multiple family meals; (3) clinician assistance preparing and supervising meals with the adolescent (e.g., at school, restaurants), meal planning, and grocery shopping; (4) clinician attendance at medical appointments with the family to ensure continuity of care and consistent messaging across providers; (5) cultural adaptations to address financial challenges and racial and ethnic diversity of presenting families (including availability of providers who were fluent in Spanish and already familiar with the unique barriers and cultural contexts experienced by families presenting for treatment in their respective agencies); and (6) introduction of non-FBT elements (distress tolerance and emotion regulation skills) to meet the needs of the complex patient population and supplement family sessions when caregivers were unavailable for multiple hours of therapy each week. The details of each of these adaptions are described using the FRAME framework to provide context about the nature of each adaptation, why and by whom it was made, and how and when it was implemented (see Table 1).

**Table 1:** Adaptations to family-based treatment (FBT) delivered in home-based settings

Adaptation	What is modified?	When, who involved in the modification, and preservation of core components?	Goal	Reasons
Delivery of intervention in the home-based (or community-based) setting, rather than the office	a. Contextual modification at the organizational level - Setting (intervention delivered in a different setting–home-based versus office-based care) - Personnel (intervention delivered by clinicians who exclusively deliver intensive home-based care services) b. Content modification at the organizational level - Condensing and intensifying dose (30–96 h over 10–16 weeks, rather than 16–20 h over 6–12 months)	Proactive adaptation made in the planning/pre-implementation phase in collaboration with researchers and program manager(s) a. Contextual modification: Preserves the core elements and functions (i.e., FBT clinician can still work to empower the family within the home) b. Content modification: Does not change core elements and functions, but rather the way in which core elements (e.g., caregiver empowerment) are delivered	a. Contextual modification: - Increase reach/engagement - Increase retention - Improve feasibility - Improve fit with recipients, including cultural appropriateness - Increase satisfaction b. Content modification: - Improve clinical outcome	a. Contextual modification: - Recipient: mental health stigma impacting willingness to present for office-based care - Recipient: access to resources (including time, transportation) b. Content modification: - Sociopolitical: existing regulations requiring 3–6 h of treatment per week for home-based services reimbursement
Additional opportunities for family meal coaching	Content modification at the organizational level (in response to intensifying dose for home-based care) - Adding elements (additional meals and greater opportunities for meal coaching with more diverse goals than the "one more bite" intervention) - Tailoring/refining the intervention (therapist may partake in meal, if culturally appropriate)	Proactive adaptation made in the planning/ pre-implementation phase in collaboration with researchers and program manager(s) Preserves the core elements and functions (i.e., FBT clinician can still work to empower the family within the home)	- Increase engagement - Improve fit with recipients, including cultural appropriateness - Increase satisfaction - Improve clinical outcomes	- Sociopolitical: existing regulations requiring 3–6 h of treatment per week for home-based services reimbursement - Provider: prior experience providing support to families in relevant daily activities as part of treatment - Recipient: caregivers often request more meal support; when families are "stuck" in FBT, caregivers often cite significant struggles in supporting their child at meals, and report that having a meal in the office setting does not approximate their experience in the home setting; may feel offended if therapist does not "join" them in eating
Additional services to support family in grocery shopping and meal preparation	Content modification at the organizational level (in response to intensifying dose for home-based care) - Adding elements (support prior to meals)	Proactive adaptation made in the planning/ pre-implementation phase in collaboration with researchers and program manager(s) Preserves the core elements and functions (i.e., FBT clinician can still work to empower the family by asking how they would like to proceed at every step, reflecting on what they are doing, and reinforcing their efforts when effective)	- Increase engagement - Increase retention - Improve fit with recipients, including cultural and socioeconomic appropriateness - Improve clinical outcomes - Increase satisfaction	- Sociopolitical: existing regulations requiring 3–6 h per week of treatment for home-based services reimbursement - Provider: prior experience providing support to families in relevant daily activities as part of treatment - Recipient: caregivers may benefit from additional education about high density food options and preparations that fit within their budget
Direct case coordination with school and other treatment providers	Content modification at the organizational level (in response to typical role of home-based provider) - Adding elements (therapist attends medical appointments, directly communicates with school to facilitate staff support of student)	Proactive adaptation made in the planning/pre-implementation phase in collaboration with researchers and program manager(s) Preserves the core elements and functions (i.e., FBT clinician can provide additional direct coordination with other treatment providers and school staff without undermining caregiver empowerment)	- Improve fit with recipients, taking into account family’s capacity to effectively advocate for their child in the context of potential language barriers and racial and ethnic or socioeconomic discrimination - Improve clinical outcomes through improved coordination and alignment among the treatment team, given the frequency of misaligned messaging to families that would complicate treatment - Increase satisfaction	- Organizational setting: service structure is one that supports high levels of coordination with other systems to provide intensive support to family - Sociopolitical: existing regulations requiring 3–6 h per week of treatment for home-based services reimbursement - Provider: prior experience providing intensive care coordination for their cases - Recipient: caregivers may not feel empowered to advocate for their child in the absence of prior experience doing so, and/or in systems that may not have been historically responsive to their concerns
Incorporation of individual sessions with the adolescent focused on distress tolerance and coping skills	Content modification at the organizational level (in response to intensifying dose for home-based care) - Adding elements (individual sessions with adolescent, evidence-based skill building)	Proactive adaptation made in the planning/pre-implementation phase in collaboration with researchers and program manager(s) Preserves the core elements and functions (i.e., FBT clinician can still work to empower the family within the home)	- Improve acceptability/fit with recipients - Align intervention with cultural values/norms (i.e., families who may highly value adolescent independence) - Improve clinical outcomes	- Sociopolitical: existing regulations requiring 3–6 h per week of treatment for home-based services reimbursement - Provider: prefer to deliver interventions in which there is a significant individual therapy component - Recipient: adolescents and their caregivers often request more skills for the adolescent in the context of FBT

## Results

FBT is a phasic intervention with sessions occurring weekly during Phase I, bi-weekly in Phase II, and monthly in Phase III [5]. Phase I focuses on uniting and empowering caregivers to take control of the adolescent’s eating- and weight-related behaviors. The clinician attempts to absolve caregivers of blame related to the etiology of AN, externalize the illness from the adolescent, reinforce positive aspects of parenting, and help caregivers develop and implement a plan for re-feeding the adolescent. Phases II and III focus on transitioning control of eating back to the adolescent, as developmentally appropriate, and shifting to supporting general adolescent development, once weight restoration is nearly complete.

Applying the structure and principles of FBT in the home required several considerations, the foremost of which concerned the altered dose of treatment (adaptation #1). The decision to adapt the dose of FBT interventions applied in the home was primarily driven by the high acuity of patients presenting in the community settings, who were frequently stepping down from or on a waiting list for a higher level of care (typically hospital-based treatment) and thus required more intensive services than the traditional once-per week, 50-min session offered through standard outpatient care. This adaptation allows for a higher level of care (i.e., similar to an intensive outpatient program) to be delivered *within the home* that centers and empowers caregivers, rather than needing to “outsource” refeeding to a more resource intensive (e.g., in terms of cost, staffing) external program that removes adolescents from their families. Enhancing the dose of treatment also addressed structural/pragmatic constraints, as third-party payors in Rhode Island reimburse for home-based services only if delivered at a minimum frequency/duration (usually 2–3 sessions per week, each lasting 1–2 h).Yet, since most caregivers are not available for several hours of psychotherapy per week, session content was adapted to meet the needs of the more complex and/or severe patient population and to align with time requirements of the home-based model. We adopted an approach developed by other groups that integrates skills (e.g., distress tolerance, coping skills) to help adolescents better tolerate FBT interventions while not undermining re-feeding attempts [49]. These skills were delivered in the context of individual therapy, with the goal of improving acceptability for clients and their families, who often request additional support for the adolescent (adaptation #6), while not compromising the core tenets of FBT (e.g., parental empowerment).

An additional adaptation relates to the family meal, typically completed at a single session during Phase I, the goal of which is to allow the therapist to observe family meal patterns, identify ways in which AN is sabotaging caregivers’ re-feeding attempts, and help families experience success with re-feeding (i.e., encouraging the adolescent to eat “one more bite” than planned). Given the intensified dose of home-based treatment, and the relative ease of providing meals in home vs. office-based settings, family meals may recur multiple times (adaptation #2) during home-based treatment (an adaptation that has been undertaken by other research groups; [50]), allowing goals to evolve with treatment progress (e.g., shifting from “one.

more bite” to meal completion). Conducting family meals in the home may provide unique opportunities for clinicians to support/model meal preparation techniques and observe mealtime behaviors and interaction patterns that might not emerge in office-based settings (adaptation #3) (Table 2).

**Table 2:** Adaptations in action: multiple family meals

Sarah was a 17-year-old, White, female-identifying patient with anorexia nervosa, restricting subtype. During an 8-week course of home-based FBT, the therapist conducted weekly family meals to scaffold support for the parents in overseeing reintroduction of regular meals and snacks to support Sarah’s return to her premorbid weight trajectory. Initially, Sarah’s mother, Diane, focused on supporting Sarah in taking “one more bite” than intended, which later shifted to 100% meal completion. Shepherd’s pie was a meal that was typically served in Sarah’s home, and one that Sarah had enjoyed prior to the onset of the eating disorder. Before starting FBT, if Sarah had a challenging time with a meal, the meal was typically not presented again in favor of meals that were anticipated to be better tolerated by the eating disorder (e.g., vegetable-based soups and salads). During home-based FBT, Diane instead began to repeatedly present meals that Sarah struggled with and provided support by balancing validation (“I know this is hard for you”) and providing clear instructions to consume the meal (“Please move your plate closer to you”). During the first family meal, Sarah refused to face the table, and the therapist encouraged Diane to provide direct prompts to support Sarah in eating her meal. At this recommendation, Diane prompted Sarah to turn her chair towards the table, place her feet on the floor, and pick up her fork. During this meal, Sarah was able to take three bites of the shepherd’s pie, despite having initially stated that she was not going to touch the meal. Although Diane was hesitant to present this meal again, the therapist encouraged her to try again at the next session. The following week, when the meal was presented again and Sarah became distressed during the meal, the therapist was able to work with Diane to identify additional tools, such as allowing a time-limited break before having Sarah return to the meal. Diane stepped out of the dining room with Sarah and gave her 5 min to use distress tolerance skills before returning to the table. Following this intervention, Sarah was able to complete 50% of the meal. Each family meal involved trying new strategies and reinforcing those that had worked previously to help Sarah complete her meal. The therapist was also able to support Diane in minimizing negotiations with the eating disorder, and in maintaining boundaries (e.g., following through with consequences outlined ahead of time surrounding meal noncompletion) that initially led to an increase in eating disorder pushback (e.g., verbal aggression towards her mother). The therapist was also able to decrease the level of intervention, such that Diane was generally encouraging meal completion of her own volition using previously successful strategies. By the end of home-based FBT, Sarah was consistently completing 100% of her meal during in-session family meals	

As home-based care is not confined to a specific setting, clinicians may support treatment-related activities in the community, school, and healthcare settings. This allows scaffolding of support for families who may be overburdened with financial, social, and/or caregiving responsibilities and struggle to prepare and monitor all meals and snacks as prescribed in Phase I. Indeed, even in standard outpatient FBT, caregivers are charged with providing a level of support and supervision for the adolescent that approximates what is provided in a hospital-based setting. As many of the families presenting to the partner agencies are unable to provide this level of support and either cannot access or do not qualify for hospital-based care, the home-based clinician is able to fill in some of the gaps in meal supervision, while still maintaining caregivers at the forefront of refeeding and empowering them to take on the highest level of oversight that is feasible without needing to “outsource” refeeding altogether to a costly, resource intensive hospital-based program. In keeping with this approach, clinicians may assist with grocery shopping to provide psychoeducation around food choices that are most effective for re-feeding; accompany adolescents during supervised exercise; and/or provide meal supervision when the family is unable to do so, for example, in school when a qualified guidance counselor or school nurse is not available (adaptation #3) (Table 3).

**Table 3:** Adaptations in action: Clinician assistance meal planning and grocery shopping; cultural tailoring to address racial and ethnic diversity and financial challenges

Mia was a 15-year-old, Latina, female-identifying patient with anorexia nervosa, binge/purge subtype. Her parents divorced when she was a toddler, and her mother, Marisol, retained primary custody of Mia and her brother (age 12) throughout their lives. Her father had returned to his native country shortly after the divorce and had very little contact with the family since that time. From the start of treatment, Marisol expressed concerns about participating in home-based FBT as a single parent living on a single income. In particular, she described worrying that presenting foods that Mia would refuse to eat, or might purge afterwards, felt like “taking food out of [younger brother’s] mouth.” The therapist examined the family’s food supply and helped Marisol set weekly goals and menus that fit within their budget and their family’s cultural food preferences. During Phase I, the therapist would occasionally grocery shop for the family (using a list generated collaboratively with Marisol) to help alleviate caregiver burden. This enabled the therapist to generate a running list of food items that were both energy dense and low cost that Marisol could use to plan meals each week. To validate the family’s financial concerns while avoiding negotiation with the eating disorder, the therapist and Marisol collaboratively agreed that Marisol would serve at least 1–2 meals per week consisting of a food item the family (including Mia) had typically enjoyed before the onset of the eating disorder. The family gradually moved from presenting food items individually (so that any food Mia didn’t eat could be saved for a later meal) to serving multi-ingredient meals with the expectation that Mia would eat all the food she was served. As Mia transitioned to Phase II and became more involved in selecting meal and snack options, the therapist had the opportunity to accompany Mia and Marisol to the grocery store. During this outing, the therapist witnessed Mia experiencing intense anxiety (described by her as “paralysis”) when choosing between two different food options, one a more palatable, energy dense item, and the other a lower-calorie version. The therapist worked with Marisol to quickly generate and implement a plan for how to support Mia in the moment. Marisol coached Mia to put the items down and use relaxation techniques (e.g., diaphragmatic breathing) until her emotional distress returned to a tolerable level. The family then returned to the food items and Marisol instructed Mia to choose the first thing that came to mind without being filtered by her eating disorder. By the end of home-based FBT, Marisol was routinely completing her meals and had tried a favorite food item (beef empanadas) that she hadn’t eaten since before the onset of her eating disorder	

Home-based clinicians may also accompany families to medical appointments to facilitate communication within a multidisciplinary team, support consistent messaging among providers, help the family process changes in weight and other health indicators, and/or model effective communication with healthcare providers (adaptation #4). Given that a central tenet of FBT is empowering caregivers to take control of the adolescent’s eating- and weight-related behaviors, any convoluted or inconsistent messaging among team members (e.g., diverging goal treatment weights, physical activity clearance in the absence of unanimous agreement among providers) has the potential to undermine parental empowerment, cause confusion among caregivers, and fracture the treatment team. This is especially problematic if treatment recommendations are delivered in a family’s non-native language. Thus, having Spanish-speaking therapists available was especially helpful during medical appointments, as they could translate more nuanced concepts (e.g., externalization of the illness) in a way that both the family and the provider could understand. An additional challenge in FBT is coordinating care across multiple healthcare providers, particularly when these providers have little or no training in eating disorders or FBT (e.g., [51]) and they work in distinct settings. This adaptation filled a dual purpose of enhancing acceptability and meeting dose requirements for home-based treatment, while maintaining empowerment among caregivers (Table 4).

**Table 4:** Adaptations in action: Clinician attendance at medical appointments with the family

Kyle was a 14-year-old, multiracial, male-identifying patient with atypical anorexia nervosa who completed a 12-week course of home-based FBT. During the first few sessions, the family described having recently learned about Kyle’s patterns of engaging in compulsive and secretive exercise, which the therapist conceptualized as impeding weight gain and further strengthening Kyle’s eating disorder cognitions. The family was initially hesitant to curtail exercise entirely, as it “helps him cope with negative emotions” and they “don’t want to take away all his coping skills.” Kyle had recently increased his energy intake, which further reinforced the family’s belief that his exercise patterns would not be a significant impediment to future weight gain. His therapist had the opportunity to spend some of their allotted therapy hours attending medical appointments with Kyle and his family. This helped to streamline communication with the outpatient eating disorders clinic where he was being seen for ongoing medical monitoring, and unify the messages conveyed by both the FBT therapist and the medical team. For example, during one medical appointment, Kyle’s physician discussed the impact compulsive exercise behaviors could have on Kyle’s bone density (exacerbated by his already low bone density and increased risk for bone fractures) as well as the worsening of electrolyte imbalances. Kyle’s medical provider supported the therapist’s recommendation of complete exercise cessation until Kyle was able to weight restore enough to support increased movement. Following the medical appointment, the family expressed the belief that addressing certain aspects of Kyle’s exercise behaviors (e.g., no longer allowing Kyle to take the dog for daily walks) would resolve the medical provider’s concerns. The therapist reiterated the messages that the medical provider had communicated as additional evidence that more close monitoring of activity patterns and complete cessation of exercise was necessary. The therapist was also able to help parents identify behaviors that the family had not previously considered problematic, such as excessive movement and standing within the home. After several joint medical appointments and FBT sessions focused on limiting exercise alongside increasing energy intake, the family described feeling disappointed that Kyle had not gained as much weight as they had expected. In between sessions, they independently searched Kyle’s phone and found that he had set a recurring alarm in the middle of the night that he was using to wake up and exercise in his room. They described feeling like a “light bulb had turned on” in considering the covert nature of the eating disorder, and how much of a hold it had on Kyle’s functioning. In response, they placed an air mattress in their room for Kyle to sleep on to monitor his activity at night. They continued to closely monitor and limit his movement until he was consistently gaining weight, at which time they gradually reintroduced low-intensity activity such as resuming dog walks in the evening	

Finally, improving fit of FBT interventions for families that typically presented for treatment at our partnering agencies required cultural adaptations (adaptation #5). Traditional FBT incorporates many elements of a culturally sensitive approach, including deferring to the family as experts on their child, working within each family's unique strengths and limitations to develop a relevant and feasible re-feeding plan, encouraging appropriate intergenerational boundaries, discussing how differing cultural values regarding body shape/weight may impact re-feeding and familial communication patterns, framing treatment goals to be consistent with family values (e.g., focusing on shared meals as a way to increase family time), discussing how acculturative stress and intergenerational conflict may impact treatment [52], and respecting family traditions and norms (e.g., incorporation of ethnic foods, role of religion and prayer in treatment). Specific cultural adaptations embedded within FBT interventions delivered in the home include involving other important family members who may participate in family meals and working directly with families within the family’s budget to create and implement an energy-dense meal plan (Table 5).

**Table 5:** Core elements of family-based treatment (FBT) for restrictive eating disorders delivered in the office-based and home-based settings

	Core functions and forms	Office-based FBT	Home-based FBT
Participants	Support parental empowerment and leverage family support	Entire family (including siblings and extended family members living in the home) attends each session	Family attends a portion of sessions with remaining sessions devoted to individual work with the adolescent on distress tolerance and coping skills
Treatment dose	Support parental empowerment while simultaneously supporting the needs of the adolescent	Weekly (Phase I), bi-weekly (Phase II), or monthly (Phase III) sessions lasting 45–50 min each, for a total of 16–20 h of treatment over 6–12 months	Multiple sessions per week totaling 3–6 h of weekly clinical care for the family, for a total of 30–96 h of treatment over 10–16 weeks
Family meal	Identify ways in which the eating disorder is interfering with caregiver attempts to refeed; allow caregivers to experience success in refeeding *or* highlight the difficulties in refeeding that lay ahead	Occurs once at the beginning of treatment with entire family Constrained by limits of office-based setting (e.g., limited dining space, utensils, appliances) Therapist refrains from eating	Occurs multiple times, often weekly Entire family may participate, or therapist may provide one-on-one meal coaching to the adolescent Conducted in the family home or in the community, with typical amenities available in those settings Therapist may choose to partake in the meal when culturally appropriate
Gathering information on family interactions related to food/home food environment	Problem-solve ways to help caregivers effectively and efficiently refeed the adolescent	Occurs through focused questioning of caregivers and detailed recall of the prior week(s)	Therapist directly observes interactions through multiple family meals Therapist directly assesses the home food environment (e.g., surveys the pantry, observes meal preparation, grocery shops with adolescent and/or caregivers)
Coordination of care	Ensure consistent messaging among treatment providers; facilitate implementation of FBT across multiple settings	Communication with medical team usually occurs via phone if medical team is offsite, or occurs in the context of medical rounds when team is onsite Therapist may encourage family to seek special services, provide supporting documentation to school	Therapist attends medical appointments with the family Therapist works closely with school (including in-person interactions) to provide psychoeducation regarding meal supervision, provision of other support services

## Discussion

Applying FBT interventions in the home presents challenges, some of which are general to any home-based treatment and some of which are specific to the application of FBT interventions. Of the challenges more general to home-based treatment, increased clinician time/travel demands (which could compound the demanding nature of standard office-based FBT; [53]), difficulties maintaining patient privacy/confidentiality, dealing with distractions (e.g., pets), and clinician safety are the most common. These factors may contribute to burnout, affecting quality of care/adherence and retention (for both clinicians and caregivers); therefore, mitigation strategies such as resilience-building interventions and regular peer supervision/consultation are critical [54]. Furthermore, because of its resource-intensive nature, applying FBT interventions in the home may be most appropriate for families that are under-resourced and have either limited or no access to higher levels of care (e.g., due to distance from specialty treatment centers and inability to transport the ill adolescent; lack of adequate insurance coverage for hospital-based care; lengthy waitlists for local centers), although research is needed to understand for whom this approach is warranted.

Challenges specific to application of FBT principles include the high level of clinician involvement which, while designed to minimize stress on the family system, may undermine caregiver empowerment/efficacy. For example, clinicians must take care not to disempower caregivers during family meals by taking too active a role in food preparation/supervision. Relatedly, although office-based FBT stipulates that the clinician not partake in family meals, delivering FBT interventions in the home setting allows the clinician to join with the family in a different way, such that clinicians may opt to partake in family meals when declining to eat with the family could limit rapport-building and trust. In addition, involvement of multiple clinicians (often trained at the master’s level, which may necessitate additional specialized training and/or supervision) requires regular communication among the treatment team to ensure consistent messaging. Finally, in our experience, adolescents seeking home-based care often present with multiple comorbidities and complicating factors such as child protective services involvement and/or undocumented immigration status. Therefore, thorough training and ongoing supervision/fidelity monitoring is imperative.

## Conclusions

Applying FBT interventions in the home may address some of the barriers that limit scale and treatment outcome, including challenges with access and transfer of treatment skills. Delivering FBT interventions in the home has benefits of providing additional modeling, scaffolding, and support for families who might not otherwise be able to engage in FBT and allowing opportunities for clinicians to tailor treatment to a family’s unique environment and build rapport, trust, and comfort on their “home turf,” which may be particularly impactful for those reticent to engage with treatment services. Since this approach is more resource intensive than traditional outpatient care and telehealth, it may be most appropriate for individuals with AN who require more intensive care than traditional, weekly outpatient therapy provides (e.g., those at high risk of rehospitalization due to refractory illness presentation, failure in outpatient treatment; [55]) or for families who have fewer resources and need additional support to provide adequate adult supervision of meals. Although evidence-based interventions for other mental health conditions have been successfully deployed in the home-based setting to improve reach and efficacy/effectiveness, home-based treatment for eating disorders is in its infancy, marking this as a novel and promising research direction which fills a significant gap in the field.

Research is currently underway to test the effectiveness of this approach within the context of an effectiveness-implementation RCT comparing a home-based FBT-informed approach to home-based “treatment as usual” [47]. Challenges encountered in our pilot work, that we are addressing in our current RCT, include issues related to the patient population (e.g., high levels of food insecurity; limited time and resources for caregivers to devote to refeeding), systemic barriers (e.g., lack of FBT-trained providers in the community to whom to refer for outpatient treatment following completion of home-based care), and organizational constraints (e.g., high clinician turnover, especially in the context of the COVID-19 pandemic; difficulties communicating with multidisciplinary treatment providers located in other settings; [46]). Ultimately, the goal of our research is to expand access to high-quality, evidence-based care for eating disorders in community settings (particularly for families that would otherwise likely receive suboptimal or no care) through thoughtful and targeted adaptations as needed. Plans for future studies expanding the reach of home-based services to include other evidence-informed treatment models for pediatric eating disorders besides FBT (e.g., enhanced cognitive behavioral therapy), and identifying families for whom this resource intensive approach is most appropriate.

## Data Availability

Data sharing is not applicable to this article as no datasets were generated or analyzed during the current study.
